# Spinal Injuries in Children

**DOI:** 10.3389/fneur.2012.00096

**Published:** 2012-07-26

**Authors:** Saumyajit Basu

**Affiliations:** ^1^Neurosciences Division, Park ClinicKolkata, India

**Keywords:** spinal injury, pediatric spinal injury, spinal fractures in children, pediatric injury, paediatric cervical spine injury, atlanto axial injuries in children, thoracolumbar fractures in children

## Abstract

About 5% of spinal injuries occur in children – however the consequences to the society are devastating, all the more so because the cervical spine is more commonly affected. Anatomical differences with adults along with the inherent elasticity of the pediatric spine, makes these injuries a biomechanically separate entity. Hence clinical manifestations are unique, one of which is the Spinal Cord Injury Without Radiological Abnormality. With the advent of high quality MRI and CT scan along with digital X-ray, it is now possible to exactly delineate the anatomical location, geometrical configuration, and the pathological extent of the injury. This has improved the management strategies of these unfortunate children and the role of surgical stabilization in unstable injuries can be more sharply defined. However these patients should be followed up diligently because of the recognized long term complications of spinal deformity and syringomyelia.

Spinal injuries in children is a separate entity, quite different from its adult counterpart due to wide differences with regards to anthropometrics, biomechanics, injury patterns, clinical presentation, imaging analysis, and management principles. Literature addressing these issues was relatively sparse historically. However in more recent years, different aspects of the pediatric spine have been better appreciated and hence there has been a plethora of publications on this area. The typical injuries occurring in children include occipito-atlantal or atlanto-axial dissociation (Figure 1), atlanto-axial rotary subluxation (Figure 2), spinal cord injury without radiological abnormality (Figure 3), and multiple thoracic compression fracture.

**Figure 1:**
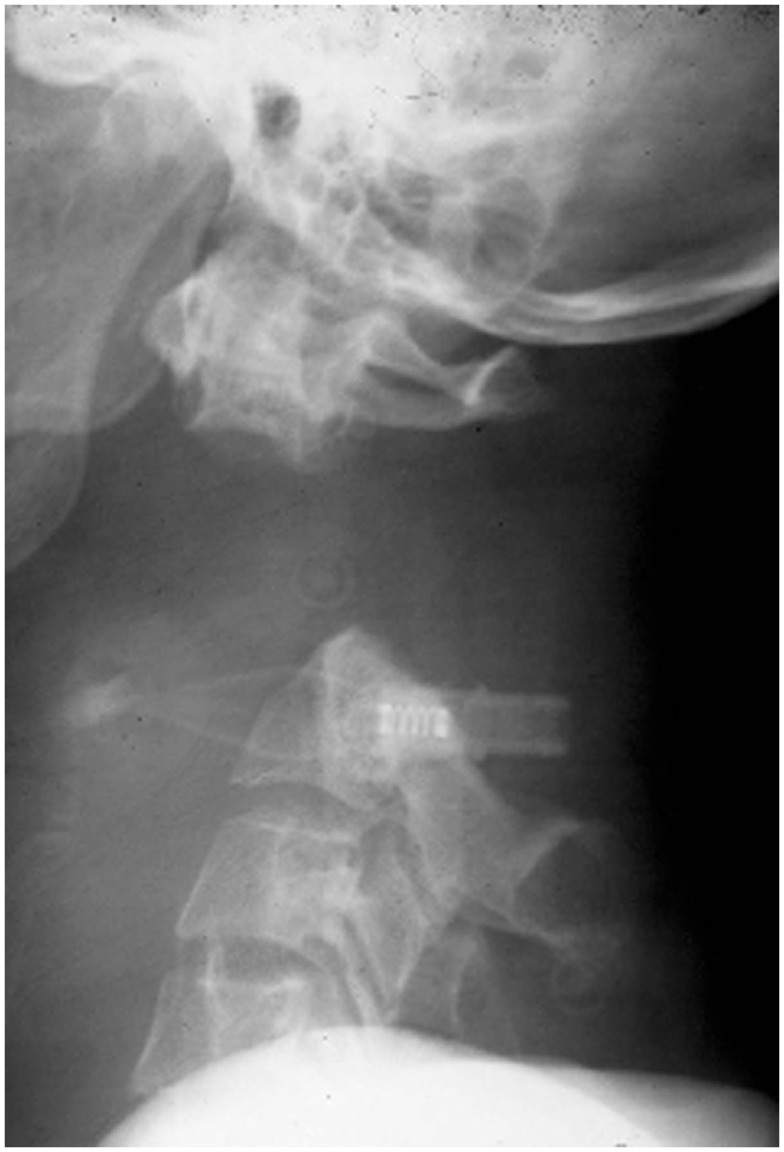
**Atlanto-axial dissociation – potentially lethal injury**.

**Figure 2:**
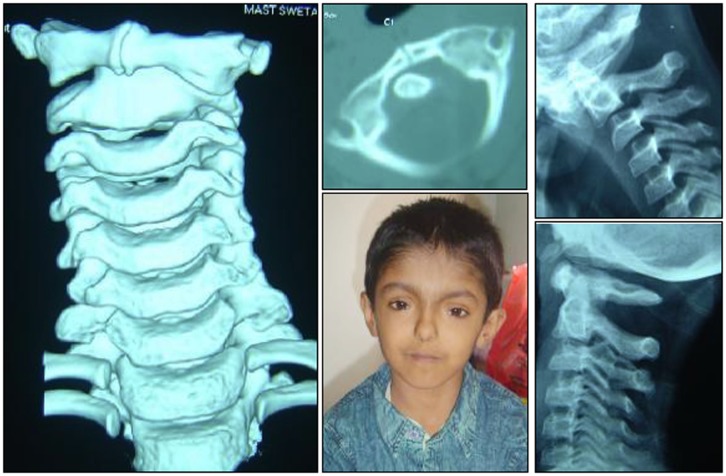
**Atlanto-axial rotary subluxation – note the absence of instability in flexion/extension radiographs though there is a slight subluxation at C2/3 which is normal**. The torticollis is characteristic.

**Figure 3:**
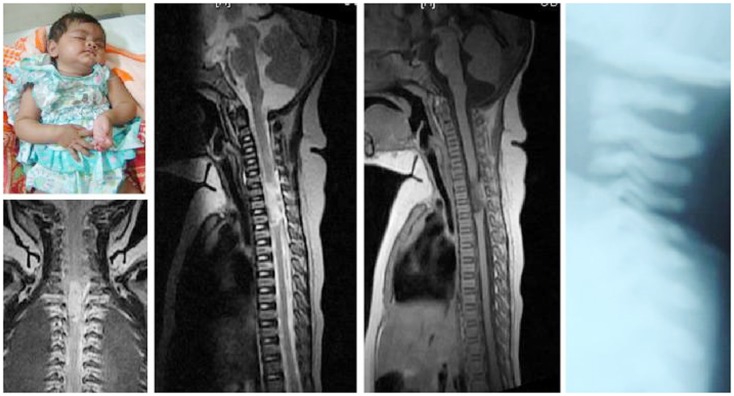
**Spinal cord injury without radiological abnormality – note the extensive cord contusion in the cervicodorsal region without any osseous or discoligamentous injury**. The flail left hand is well appreciable.

## Epidemiology

The incidence of spinal injuries in children is between 2.7 and 9% of spinal injuries (Ruge et al., 1988; Hamilton and Myles, 1992; Osenbach and Menezes, 1992) and 40–60% occur in the cervical spine (Hadley et al., 1988; Hamilton and Myles, 1992). The upper cervical area was twice as frequently injured in the younger child while thoracolumbar junctional injuries are more common in the older child. Lower cervical injuries are equal to both. In a study by Ruge et al. (1988) it was observed that children younger than 3 years represent a distinct group due to a higher incidence of C1–2 injuries, girls being more commonly affected than boys and a lesser need for surgical stabilization. The pattern of spinal injury in children is related to age and also the mechanism of injury. While traffic-related incidents are a leading cause of injury across all age groups, older children, particularly boys, sustain spinal trauma in sporting and recreational activities (Bilston and Brown, 2007).

Motor vehicle accidents are the most common causative factor and falls, obstetrical causes, sport injuries including diving accidents, and child abuse are the other important causes (Stern and Rand, 1959; Kewalramani and Tori, 1980). In neonates, the common cause of cervical injury is obstetrical complications. Spinal cord injuries occur in 1 of 60,000 births (Vogel, 1997). Important features of birth related injuries include apnea, flaccid quadriplegia in a patient with breech delivery or with the use of forceps. Death is common in such circumstances.

## Related Anatomy

### Applied embroyology

**Figure 4:**
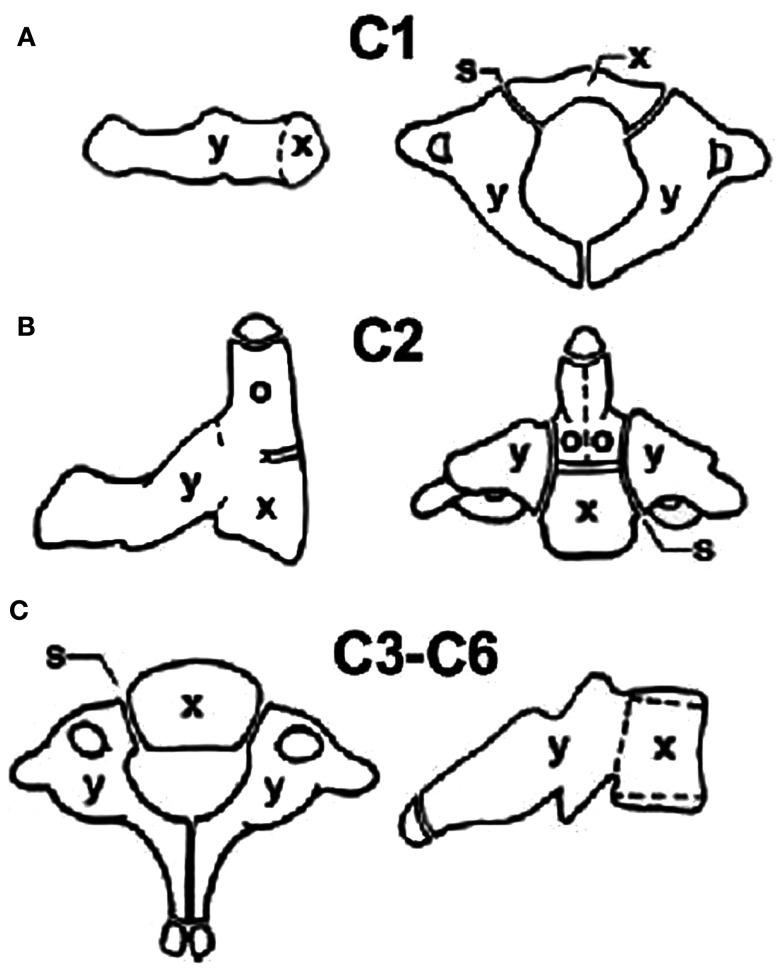
**Centers of ossification for the cervical spine: (A) Atlas, (B) Axis, and (C) C3 to C6**.

The developing spine is unique in the fact that ossifying cartilages are present and these ossification centers are joined by synchondrosis which may be mistaken for fractures (Figure 4). The atlas forms from three centers – one each of the lateral masses and one for the anterior arch (which may appear at 1 year of age). Fusion of all three centers of ossification is completed by 7 years but the midline of the posterior arch might still be un-fused and appear like a fracture because of its bifid appearance. The axis has one center for the body (centrum), one each for the posterior arches, and two for the odontoid which gets fused before birth. The odontoid is separated from the body by a cartilaginous physis which is positioned below the level of the C1/C2 facet joints and is mistaken for a fracture till the age of 5–7 years, at which they fuse. It must be realized however that the common type II fracture of the odontoid is at the level of the waist of the odontoid and hence is above the facet joints of C1/C2. The tip of the odontoid has a separate small ossification center (ossiculum terminale) appears at 7 years and fuses to the dens at 12 years. The rest of the cervical vertebrae as well as the thoracic/lumbar vertebrae follow a similar pattern with one ossification center for the body and one each for the posterior arches and lateral masses, which unite in the midline between 2–4 years age and the neurocentral synchondrosis closes at 3–6 years.

The thoracolumbar injuries in children often presents with multiple levels of fractures of the endplate, the superior endplate being more common. In the thoracolumbar spine, childhood injuries have a potential for physeal separations other than the standard osseous and ligamentous injuries in the adults. Disk spaces are usually not disrupted in the immature spine (Aufdermaur, 1974); rather cartilaginous endplate separation does occur in physeal injuries which is akin to Salter–Harris Type I fractures of long bone. If the severity of the trauma is good enough the injury traverses through the physis into the posterior elements and might result in dislocation in contrast to adult injuries leading to dislocation where the separation is through the disk or the vertebral body.

## Biomechanics

The pediatric spine is more elastic than that of adults especially below 8 years (Herkowitz and Rothman, 1984) and it has been shown in neonatal cadavers that the vertebral column could stretch by 2′′ without disruption whereas the spinal cord could only stretch by 0.25′′ (Leventhal, 1960). Hence trauma to the spine in young children can produce neural damage much earlier to musculoskeletal injury. Three factors are responsible for this intrinsic elasticity of the vertebral column in a child. Firstly the facet joints are more shallow and horizontal in children (Cattell and Filtzer, 1965), allowing some degree of slippage. Secondly ligaments and joint capsule are more stretchable (Fesmire and Luten, 1989) leading to what is known as *pseudosubluxation*. Thirdly absent uncinate processes (the joints of Luschka ossify at 7 years age, after which they contribute to stability) and weak nuchal muscles also lend more flexibility (Townsend and Rowe, 1952; Table 1). Anterior wedging of vertebral bodies and ill-developed spinous/transverse processes (decreasing the stabilizing effects of paraspinal muscles) may be additional reasons for the hyper-mobility.

**Table 1:** **Anatomical differences – pediatric and adult spine**.

No.	Structure	Difference with adults
1	Facet joints	More shallow and horizontal
2	Ligaments/capsule	More stretchable without tearing
3	Uncinate process	Absent in children
4	Bodies	Wedge shaped
5	Spinous process	Less developed

Children below 8 years have a relatively large and heavy head compared with the body which shifts the fulcrum of movement to the upper cervical spine with the maximum movement at C2/3. With growing age, at about 5–6 years, it shifts to C3/4 and in adolescents it shifts to C5/6 as in adults (Townsend and Rowe, 1952). This explains the epidemiological finding that the majority of spinal injuries occur between C0 and C2 in young children whereas older children, like adults have their injuries more commonly in the subaxial cervical spine (Hadley et al., 1988).

**Figure 5:**
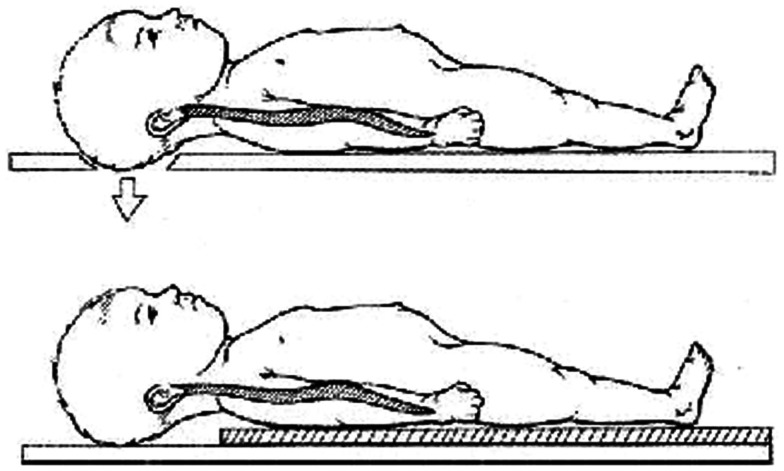
**The large head of children makes it mandatory to place a board of 25 mm beneath the trunk or create an occipital recess for all spinal boards to be used for transport of children with spinal injury**.

It is to be remembered that the other consequence of a large head in children is the natural kyphosis which occurs when the child is lying supine as on a firm backboard – in a setting of spinal trauma, this might worsen the neurodeficit and hence either the torso should be elevated (by about 25 mm) or a recess for the occiput should be created (Herzenberg et al., 1989; Figure 5).

## Spinal Cord Injury without Radiological Abnormality

Spinal Cord Injury Without Radiological Abnormality (SCIWORA; Pang and Wilberger, 1982) occur commonly in the immature spine. The incidence of traumatic myelopathy due to this condition varies, but one such review involving 617 children reported the incidence to be about 36% (Pollack, 1995). Children less than 8 years age are more predisposed to this injury because of the tenuous blood supply to the cord (Choi et al., 1986) and greater elasticity in the vertebral column than in the spinal cord (Leventhal, 1960) and younger the child, the more profound are the neurodeficits (Hadley et al., 1988; Pang and Pollack, 1989) which can also be delayed (Walsh et al., 1983). In adults this is very uncommon – to the tune of 2 per 1,000 spinal injuries (Scher, 1976). The cord damage might be due to contusion/edema/hemorrhage/hematoma/infarction. Acute thoracic disk prolapse or protrusion of cartilaginous endplate might be the causative pathology.

Clinical presentation is more often in the form of a complete spinal cord injury (SCI). Very young children (including those involved in birth injuries) might be diagnosed quite late because of involuntary reflex movements of the limbs which confuse the picture. Poor respiratory muscle effort in high thoracic injuries might lead to pulmonary complications, which might be the initial presentation. Telltale clinical signs of bruise or abrasion should immediately alert the concerned physician of a potential spinal injury. Evaluation of the distal reflexes/bladder function is hence mandatory for early diagnosis.

MRI is invaluable for assessment because it can pick up cord signal intensity changes and potential discoligamentous injury (Davis et al., 1993). However there remains a subset of these patients, where the MRI is normal. When Pang and Wilberger (1982) proposed the definition in 1982 taking into account plain X-rays – this definition is antiquated and should take into account CT and MR scans also, and strictly speaking SCIWORA, in the current scenario, should be applied to those spinal cord injuries with normal X-ray/CT scans/MRI.

These injuries are generally considered unstable and immobilization for up to 3 months is recommended (Pang and Pollack, 1989). Immobilization should be removed after confirming stability by dynamic X-rays and in those, in which MRI had documented a discoligamentous injury, delayed instability, or deformity should be looked for. Surgery has limited role in these patients except when there is deteriorating neurology in a documented extra-dural hematoma (Yngve et al., 1988). Late onset deformities in the form of kyphosis and scoliosis might have to be managed surgically.

Late manifestations of SCIWORA has also been described and a case report of an infant who had hemiparesis 6 days after a fall has been published (Kim et al., 2008). This has lead to the belief that even in minor injuries in certain cases, MRI should be done early even in the absence of gross neurodeficit. Recurrence during or after a SCIWORA has been termed as Second SCIWORA and usually occurs within 2 weeks – rarely it presents very late. Recurrence occurs in up to 17% patients (usually very young) in sports injuries and is more severe compared to the initial SCIWORA but still the prognosis is favorable (Pang and Pollack, 1989).

## Clinical Presentation

A history of trauma including motor vehicle accident, fall from height, sports related injuries, or suspicion of child abuse should alert the clinician of an impending spinal injury. Pediatric spinal injury should always be suspected if a child is brought in with unconsciousness, torticollis, and neck pain/stiffness, temporary, or fixed neurological deficits. Clinical examination should be done in great details because extracting the proper symptoms and signs is difficult in children. Signs of facial trauma, seat belt abrasions, and cradling the head with the hands should be looked for. Non-contiguous spinal injury might be present (Heilman and Riesenburger, 2001). Search should be made for other injuries to the chest, abdomen, and pelvis. For patients presenting late, one must be suspicious of children whose neck pain is not resolving within a week after injury or there is some apparent deformity like torticollis present.

Children are less likely to have neurological injury with cervical trauma although when they occur, it is usually associated with facetal dislocations with or without fractures. A unilateral dislocation is likely to produce root damage while bilateral dislocations lead to cord damage. Spinal cord injuries are often incomplete and improvement may occur very late also (Hadley et al., 1988; Ruge et al., 1988). However complete lesions usually do not recover (McPhee, 1981). Finally, as a rule of thumb, in all children admitted with head injury or unconsciousness, spinal injury has to be ruled out.

## Imaging Studies

### X-rays

**Figure 6:**
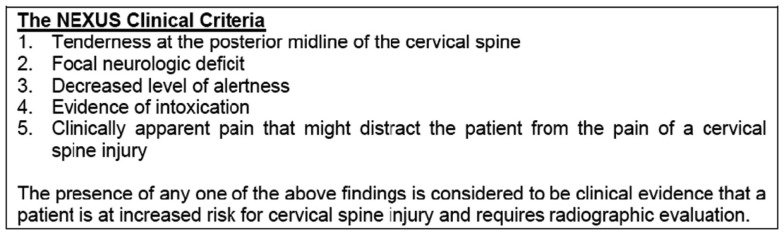
**The indications of radiographic evaluation of spinal injury in a child**.

Standard trauma series X-rays include cervical spine AP, lateral, and open-mouth views (which must include C7/T1) and thoracolumbar spine AP, lateral. The National Emergency X-Radiography Utilization Study (NEXUS) was a prospective, observational study involving 21 centers across the United States that evaluated 34,069 stable patients with blunt trauma who were at risk for cervical spine injury (Hoffman et al., 2000). Any patient who had tenderness/neurodeficit/loss of alertness/intoxication/distracting painful injury is a candidate for cervical X-rays (Figure 6). This decision instrument was validated for use in pediatric patients also (Viccellio et al., 2001).

Open-mouth views have doubtful utility in children younger than 5 years because much of the odontoid is cartilage. In some centers the odontoid view is not done in children younger than 5 years because of its questionable value (McCall et al., 2006). Active flexion/extension X-rays should be done in all patients who do not meet the NEXUS criteria and whose routine X-rays are negative. They should be done in awake, cooperative, and neurologically intact patients They should also be done if there are suspicious findings on the routine X-rays like segmental kyphosis, possible subluxation, or soft tissue swelling (Ralston et al., 2001). Dynamic X-rays are not useful if the full range of motion is not possible because of pain or muscle spasm (d’Amato, 2005). AP and lateral views of the thoracic/lumbar spine is done before further management.

**Figure 7:**
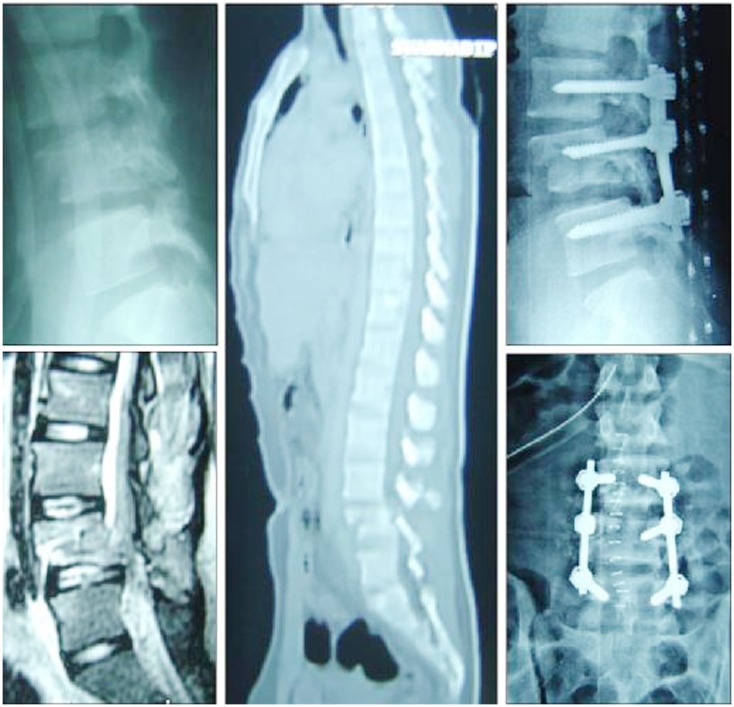
**This 15-year-old boy suffered a chance fracture of L4 with bilateral foot drop and 5 mm pedicle screws used for spinal stabilization**. Note the fusion was intentionally kept short to avoid including the sacrum.

It must be appreciated that because of the factors described in Table 1, there can be a step-off seen on the lateral cervical X-rays at C2/3 and occasionally at C3/4 also. Pseudosubluxation in the upper cervical spine in young children is considered normal (Figure 7) and 3 mm of anterior displacement was present in 40% at C2/3 and 14% at C3/4 (Cattell and Filtzer, 1965). Other than pseudosubluxation, the other neuroimaging pitfall in pediatric patients is that the synchondrosis between dens and the body of C2 is mistaken for a fracture. However, as mentioned above the physis is usually below the typical location of a type II fracture of the odontoid. The physis between the dens and the arch is also occasionally mistaken for a fracture. It is important to note that this physis is appreciable only on the oblique views while the fractures are usually appreciable on a lateral view.

The soft tissue space anterior to C2 should be 7 mm or less and anterior to C6 should be less than 14 mm. These areas may wider in a crying child. The atlanto-dens interval (ADI) should be less than 4 mm – however in very young children, up to 5 mm can be considered healthy.

## Role of CT Scans and MRI

Keeping in mind the well-accepted fact that CT scan show a better bony architecture and MR scan shows a better soft tissue anatomy, it has to be remembered that most of pediatric spinal injuries are ligamentous in nature without osseous component (Hamilton and Myles, 1992). Even in children older than 10 years, about 20% of injuries would be purely ligamentous (Viccellio et al., 2001). Hence though CT scans may be superior to plain radiographs in some aspects, they should not be used exclusively for cervical spine clearance (Schleehauf et al., 1989).

MRI can be very useful to clear obtunded, intubated, or uncooperative child (Flynn et al., 2002). If a child has persistent neurological symptoms MRI is helpful to reveal ligamentous or disk injury and of course show the neural elements in great details. MRI can also provide useful information in cases of SCI especially with regards to prognosis depending on the extent of signal intensity changes of the cord. MRI is an invaluable tool for evaluating patients with SCIWORA. Findings include spinal cord hemorrhage, transection, and ligamentous or disk injury (Davis et al., 1993).

## Management Strategies

Most pediatric spinal injuries can be treated conservatively (Birney and Hanley, 1989) and even ligamentous injury in young children can heal without surgery (Sherk et al., 1976), as opposed to adolescents and adults. However, given the hazards of prolonged immobilization in a child, especially with the halo, more and more surgeons are electively treating many of these patients surgically (Bilston and Brown, 2007).

### Conservative treatment

The main-stay of non-surgical treatment is rest and external immobilization, which, in children is difficult to fit in correctly without hindering day to day activities. A custom made Philadelphia collar for upper cervical injuries and a similar Minerva orthosis for lower cervical or cervicodorsal injuries are usually applied. Thoracolumbar orthosis include Taylor’s brace and ASH brace. Though halo-vest immobilization gives a superior fixation as proved by biomechanical studies, applying it in children can lead to more complications than adults (Baum et al., 1989), probably due to their thinner scalp and skull and reported rate of complications can be as high as 68% (Dormans et al., 1995). Pin site infections, loosening, dural, and supra-orbital nerve injury are the important complications in order of occurrence (Dormans et al., 1995). While adults and children above 5 years require 4 pins to stabilize the halo, children below 2 years require 8–10 pins (Mubarak et al., 1989) and as they grow older, fewer pins are required. The amount of torque which is required for halo brace is significantly lesser in smaller children. Traction is occasionally required to be given through the halo to restore cervical alignment and is much safer than Gardner–Wells tongs in small children. One pound of traction per cervical level should be adequate in children younger than 4 years and it should be increased to 2 lbs for those above 4 years (McCall et al., 2006).

### Surgical treatment

Surgery is usually indicated for grossly unstable injuries (especially with progressive neurodeficit), non-reducible dislocations, and progressive deformities and of course, for decompression of the neural elements. Anterior or posterior approach is best dictated by the column which is maximally disrupted – effort should be made to go through the damaged tissue rather than invade virgin tissue. Progressive deformity has been reported in patients with posterior cervical ligamentous disruption, treated with anterior cervical fusion (Stauffer and Kelly, 1977).

Twenty six relevant articles on the use of instrumentation in children were identified in a recent study by Parent et al. (2010) out of which 6 were on thoracolumbar injuries and 16 on cervical injuries, the rest being a mixed bag. All were retrospective studies – case reports or cohort studies. The 16 papers on cervical instrumentation reported satisfactory results with low complication rates. C1/C2 transarticular screws were found to be safe in a study on 67 children (Gluf and Brockmeyer, 2005) and 100% fusion rates were reported with 10.4% complication rate including two cases of vertebral artery injury. Anderson et al. (2007) has reported on the largest series comprising of 95 children who underwent cranio-vertebral stabilization and followed up 17 patients for over 5 years. He found no long term complications of adjacent level degeneration or growth arrest or instrumentation failure.

**Figure 8:**
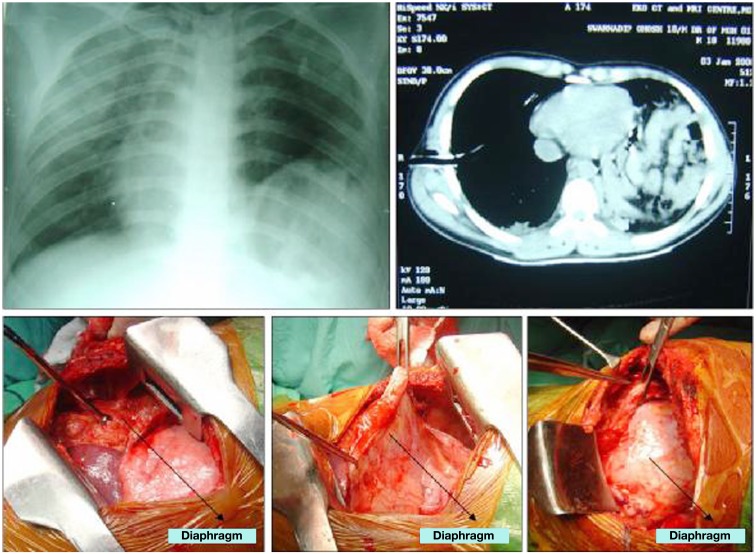
**He also had an associated diaphragmatic rupture which was surprisingly asymptomatic, being picked up on a routine chest X-ray which showed the heart shadow pushed to the right along with abnormal elevation of the left diaphragm**. CT confirmed the diagnosis and repair of the diaphragm was done in the same sitting. The left lung and the spleen was found lying side by side along with the torn diaphragm.

Pediatric population tolerates thoracolumbar spinal instrumentations in the form of pedicle screws quite well (Figures 7 and 8). Santiago et al. (2006) reported on 13 (out of 96 patients) and Dogan et al. (2007) reported 23 (of 89 patients) cases of thoracolumbar instrumented fusions for traumatic conditions without significant complications but with visible callus formation (which is hardly seen in adults). The safety and efficacy of spinal instrumentation in pediatric deformities has been well established by many studies (Hedequist et al., 2004).

Other factors including growth potential of the child, the size of implants required to be put in and the paucity of autogenous graft is an important consideration peculiar to children. For maintaining the growth potential of the spine long segment fusions are usually avoided. Pediatric implants if available is preferred. Posterior cervical lateral mass screws should be used in the older child. Pedicle screws have been proved to be safe in very young children also though smaller diameter has to be used. Adequate soft tissue cover needs to be given over bulky implants for proper healing. Iliac crest bone grafts are inadequate in small children and the rib, medial surface of the tibia or the fibula might have to be harvested for getting bone. Allograft should preferably used in compressive mode with a fixation device, typically in anterior cervical fusion, and their failure has been documented when used in a posterior construct (Koop et al., 1984).

## Late Complications of Pediatric Spinal Injuries

### Spinal deformity

Nearly all children of SCI before their growth spurt develop spinal deformities (Dearolf et al., 1990) – the younger the patient is, more is the likelihood of developing scoliosis. The curves behave like paralytic or neuromuscular scoliosis. Hence these patients should be closely followed up from the time of their SCI and bracing should be started when the cobb angle overshoots 10° to prevent the development of scoliosis and there is some evidence that if initiated before 20° it can delay the progression of deformity significantly (Mehta et al., 2004; Parent et al., 2010). Though there is some impairment of daily activities by a thoracolumbar orthosis, the benefits possibly outweigh them. In established cases of scoliosis, when the child has become an adolescent, correction of deformity by instrumentation is recommended, using the principles of correction of neuromuscular scoliosis so that restoration of sitting balance is achieved along with a strong painless spine. Frequently long segment instrumentations from the upper thoracic spine to the pelvis is done.

### Syringomyelia

Post-traumatic syringomyelia is quite common in SCI patients. There are some reports of extensive and progressive syrinx formation from the thoracolumbar spine up to the upper cervical spine. Residual kyphosis and canal stenosis after remodeling is completed has been suggested to be important factors for the development of syringomyelia. In retrospective MRI studies, 20–40% patients of focal kyphosis more than 15° and canal stenosis more that 25% have a tendency to develop syringomyelia (Abel et al., 1999). Patients usually present with increased spasticity and in some patients the neurological level of affection creeps upward. In these patients, progression of syrinx formation should be suspected. The results of surgery in the form of syringo-subarachnoid shunt or syringo-pleural shunt are unpredictable and not always very rewarding.

## Conflict of Interest Statement

The author declares that the research was conducted in the absence of any commercial or financial relationships that could be construed as a potential conflict of interest.
